# Hypothesis: a Unifying Mechanism for Nutrition and Chemicals as Lifelong Modulators of DNA Hypomethylation

**DOI:** 10.1289/ehp.0900741

**Published:** 2009-07-08

**Authors:** Duk-Hee Lee, David R. Jacobs, Miquel Porta

**Affiliations:** 1 Department of Preventive Medicine and Health Promotion Research Center, School of Medicine, Kyungpook National University, Daegu, Korea; 2 Division of Epidemiology, School of Public Health, University of Minnesota, Minneapolis, Minnesota, USA; 3 Department of Nutrition, University of Oslo, Oslo, Norway; 4 Institut Municipal d’Investigació Mèdica, and School of Medicine, Universitat Autònoma de Barcelona, Barcelona, Spain

**Keywords:** chemicals, DNA hypomethylation, epigenetics, glutathione, nutrient, persistent organic pollutants

## Abstract

**Background:**

Although both nutrition and chemicals are important environmental factors modulating epigenetic changes, they are commonly studied separately by researchers in different fields. However, these two environmental factors cannot be separated from each other in the real world because a number of chemical agents contaminate food chains.

**Objective:**

We propose a unifying mechanism that can link epigenetic alterations in relation to DNA hypomethylation due to chemical agents and to nutrient deficiency or imbalance, emphasizing the importance of an integrative approach in the field of environmental epidemiology.

**Discussion:**

Methyl groups from *S*-adenosylmethionine (SAM) are needed for DNA methylation. Diets low in sources of methyl groups can lead to global DNA hypomethylation by impairing synthesis of SAM. However, even without nutritional deficiency, enhanced need to synthesize glutathi-one (GSH) can impair synthesis of SAM and perturb DNA methylation, because the methylation cycle and the GSH synthesis pathways are biochemically linked. Exposure to environmental chemicals is a common situation in which the need for GSH synthesis is enhanced, because GSH is consumed to conjugate diverse chemicals. Given that GSH conjugation happens at any chemical dose, this hypothesis is relevant even at exposures below the high doses that cause toxicologic responses.

**Conclusion:**

At present, general populations are exposed to a large number of chemicals, each at a very low dose. Thus, DNA hypomethylation due to chemical exposure may be common in modern societies and can synergistically interact with nutrition-induced DNA hypomethylation.

Epigenetics is the study of meiotically and mitotically induced heritable changes in gene expression through DNA methylation, histone modifications, or microRNA change without actual modification in the genomic DNA sequence (Feinberg 2007; Ozanne and Constancia 2007). Because gene expression is influenced by various environmental factors, epigenetics can serve as an interface between the inherited static genome and the dynamic environment (Feinberg 2007; Ozanne and Constancia 2007), presenting immense challenges to environmental epidemiology (Porta 2008).

Whereas epigenetic dysregulation is increasingly implicated in cancer, the role of epigenetics in other complex chronic diseases, such as cardiovascular disease, type 2 diabetes, and obesity, remains largely uncharacterized. So far, research efforts on causes of epigenetic changes have focused on nutrition during pregnancy as primary influences of epigenetic patterns in childhood and adulthood (Mathers 2007). In particular, applying the theory of the fetal origin of adult diseases, low birth weight due to undernutrition is hypothesized to explain in part the currently ongoing epidemics of metabolic syndrome and type 2 diabetes—for example, in developing countries as India or China (Yajnik 2004). Preconceptional exposure to famine during the Dutch Hunger Winter of 1944–1945 was recently shown to be associated with hypomethylation of the insulin-like growth factor 2 (IGF2) gene six decades later (Heijmans et al. 2008). Because IGF2 plays a key role in mammalian growth, influencing cell division and differentiation and possibly metabolic regulation (O’Dell and Day 1998), the study provided empirical support for the hypothesis that undernutrition during pregnancy increases risk of adult-onset diseases through epigenetic modulation of gene expression (Gluckman et al. 2008).

On the other hand, evidence is also emerging that various common environmental chemical agents, including some endocrine disruptors, can affect normal developmental epigenetic processes and hence contribute to increase the risk of chronic disease in adults (Edwards and Myers 2007). Unfortunately, epigenetic changes due to these two important types of environmental factors—nutrition and chemicals—tend to be studied separately by researchers in different fields.

Because numerous chemical agents contaminate food chains, however, these two environmental factors cannot be separated from each other in the real world. Furthermore, these factors can synergistically cause epigenetic changes through a common pathway, as we hypothesize below. Environmental epidemiology is a unique study area that observes freely living human populations and tries to both disentangle and integrate complex etiopathogenic processes that involve very diverse risk factors. It is hence critical for scientific progress to better understand how nutrition and synthetic chemicals can be related.

## DNA Hypomethylation through a Unifying Mechanism

Global hypomethylation of the genome largely affects the intergenic and intronic regions of the DNA, particularly repeat sequences and transposable elements, and is believed to result in chromosomal instability and increased mutation events (Wilson et al. 2007). Regardless of tissue type, human cancers have in common both global genomic hypomethylation and focal CpG island hypo- and hypermethylation (Franco et al. 2007). In addition, global hypomethylation is also associated with other diseases such as atherosclerosis (Zaina et al. 2005). Here, we propose a unifying mechanism that can link epigenetic alterations in relation to global or focal DNA hypomethylation due to chemical agents and to nutrient deficiency or imbalance that has little been considered among epidemiologists and other researchers. We do not think that our hypothetical mechanism can explain all epigenetic mechanisms, including focal DNA hypermethylation in specific genes, although chemical exposures and nutrition deficiency or imbalance likely lead to other epigenetic changes through unknown mechanisms.

**Figure 1:**
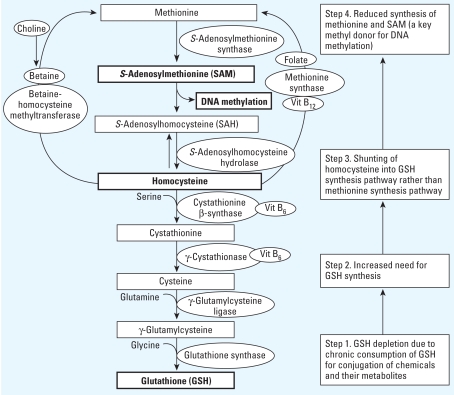
A hypothetical unifying mechanism linking DNA hypomethylation due to chemicals and nutrient deficiency or imbalance. Vit, vitamin. DNA methylation pattern can be disturbed because of depletion of GSH when it is chronically consumed for conjugation of chemicals and their metabolites. Under usual circumstances, metabolism of homocysteine contributes to both the methionine and GSH synthesis pathways. In the presence of chemicals such as persistent organic pollutants that deplete GSH, contribution to the methionine pathway may be diminished because of greater need to synthesize GSH (numbered boxes on the right).

SAM (*S*-adenosylmethionine) is a critical methyl donor for most methyltransferases that modify DNA, RNA, histones, and other proteins (Loenen 2006). Folate, methionine, betaine, choline, and vitamin B_12_ are involved in one-carbon metabolism, which includes SAM-substrated methylation (Mason 2003). Thus, diets lacking in substrates or cofactors in one-carbon metabolism can contribute to DNA hypomethylation by impairing synthesis of SAM (Davis and Uthus 2004; Selhub 2002). This methylation cycle (Figure 1, top) is very well known and frequently cited to explain relations between diet and epigenetic changes. However, even without nutritional deficiency of methyl groups, impaired synthesis of SAM and perturbed DNA methylation can happen when the need for glutathione (GSH) synthesis increases (Figure 1, bottom).

Exposure to chemical compounds may be a common situation in which humans need more GSH (Jones et al. 1995). GSH and GSH transferases have evolved as a major chemical protection against reactive xenobiotics and reactive compounds produced during the metabolism of endogenous and exogenous compounds (Ketterer et al. 1983). GSH transferases have broad and overlapping substrate specificities, which allow them to participate in the detoxification of a chemically diverse group of compounds. The most common reactions involve nucleophilic attack by GSH on electrophiles, usually epoxides of aromatic and aliphatic organic compounds (Coles and Ketterer 1990). These substrates have in common a degree of hydrophobicity and possess electrophilic centers (Coles and Ketterer 1990).

Experimental studies have reported that exposure to chemicals increased GSH content by enhancing the uptake of amino acid substrates and the activity of biosynthetic enzymes (Franco et al. 2007; Shi et al. 1994) as an adaptive mechanism against short-term exposure to chemicals. Increased need for GSH reduces the availability of homocysteine, which stands at the crossroads between the methylation cycle and the transsulfuration pathway, for use in the methylation cycle. Eventually, by shunting homocysteine into the GSH synthesis pathway, the levels of methionine and SAM would decrease (Figure 1).

If the exposure to chemicals is transient, all these disturbances can quickly return to normal without progression to the levels of GSH depletion. However, when there is prolonged exposure to chemicals, it can eventually progress to the depletion of intracellular GSH through GSH consumption by conjugation (Franco et al. 2007). Many field studies on aquatic organisms living in polluted areas have reported decreased GSH content compared with those of unpolluted areas (Cossu et al. 1997; Otto and Moon 1996). Unlike *in vitro* or animal experiments in which exposure patterns to chemicals are not similar to that of free-living humans in terms of exposure duration or number of chemicals, naturalistic field studies can give information relevant to physiologic response to background exposure to mixed xenobiotic substances.

There is direct experimental evidence that depleting GSH decreases the level of SAM in cells and leads to genomewide DNA hypomethylation (Lertratanangkoon et al. 1997). In experimental studies, the depletion of GSH is commonly induced by direct inhibition of GSH synthesis enzymes or in knockout models (Akai et al. 2007; Wu and Cederbaum 2004). However, in general populations living in chemical-contaminated societies, a more common mechanism for GSH depletion may be GSH consumption through conjugation with chemicals or their metabolites.

Once GSH depletion occurs, a vicious cycle may start. As a crucial molecule in anti-oxidant defense of the cell, the depletion of intracellular GSH causes a cascade of events that entails oxidative stress, including production of reactive oxygen/nitrogen species (Higuchi 2004). Oxidative stress can directly suppress the methylation cycle by limiting activity of folate-dependent methionine syn-thase and cobalamine (Deth et al. 2008). In addition, GSH is further consumed to conjugate reactive oxygen/nitrogen species that are generated because of oxidative stress, leading to enhanced GSH depletion.

## Background Exposure to Mixtures of Chemicals

Importantly, the theoretical soundness of our hypothesis does not require individual high-dose chemical exposure that is usually required to cause a classic toxicologic response. In fact, in many countries across the world, groups in the general population are exposed to very low doses of each chemical, often below limits currently deemed safe (Paustenbach and Galbraith 2006; Porta et al. 2008). However, GSH conjugation may happen at any dose because it is a physiologic response to excrete “foreign bodies.” For a person exposed to a single chemical at a low concentration, GSH consumption is trivial. However, if the exposure is to a large number of chemicals for a long time, GSH use is relevant and depletion can happen because of GSH conjugation.

In our modern societies, hundreds of chemicals are detected in significant subsets of the population, with concentrations of the compounds being highly correlated (Paustenbach and Galbraith 2006). Most such exposure occurs during usual daily life, implying a chronic, lifetime exposure. Thus, alterations in methylation patterns due to background exposure to mixtures of chemical agents are plausible. They may be more serious when nutritional deficiency or imbalance involved in the methylation cycle coexists.

Another important consideration is that global DNA hypomethylation accumulates progressively during aging (Fraga et al. 2007). At present, it is unclear why aging is related to global DNA hypomethylation. However, the GSH depletion could be involved in this association because GSH levels decline in a number of tissues during aging through perturbation of the catalytic efficiency of glutamate-cysteine ligase, the rate-limiting enzyme in GSH synthesis (Liu et al. 2004). At the same time as this perturbation in catalytic efficiency is occurring, the body burden of chemicals with long half-lives tends to increase with aging. Moreover, deficiencies of vitamin B_12_ and folate are common in elderly people (Koehler et al. 1997). Thus, chemical exposure, nutrition, and aging can interact with each other synergistically, finally leading to global hypomethylation.

Chemical-induced epigenetic changes can be heritable across generations (Anway et al. 2005, 2006), raising the possibility that some familial aggregation of chronic diseases may be partly related to chemical exposures in earlier generations. If confirmed in the next few years, such evidence will be a relevant example of a scientific and public health challenge to which environmental epidemiology may contribute by enabling research that effectively integrates reasoning, methods, and evidence from life course, environmental, molecular, and epigenetic epidemiology (Porta 2008).

## Persistent Organic Pollutants and Epigenetic Changes

Among chemicals putatively relevant in environmental epigenomics, persistent organic pollutants (POPs) are of particular concern because they bioaccumulate in adipose tissue throughout life (Abelsohn et al. 2002). Humans are typically exposed to a variety of POP mixtures starting *in utero*, essentially because these chemicals bioaccumulate in food chains and transfer from the mother’s dietary intake through the placenta (Abelsohn et al. 2002).

Another important feature of POPs can also lead to GSH depletion. Lipophilic xenobiotics such as POPs are secreted into the bile from the liver and are reabsorbed from the intestinal lumen into the blood circulation and back to the liver, thus undergoing entero-hepatic circulation (Jandacek and Tso 2001). This feature of POPs is one of main reasons for long half-lives of POPs in the human body (Jandacek and Tso 2001). POPs are secreted into the bile conjugated with molecules such as GSH (Bakke et al. 1990). Thus, the continuous recycling of POPs through enterohepatic circulation can lead to chronic depletion of liver GSH. Also, because there are strong correlations between serum concentrations of POPs and age in the general population (Lee et al. 2006), POPs provide examples of chemicals that can synergistically increase the risk of global hypomethylation with aging.

Global DNA hypomethylation was recently associated with high serum concentrations of POPs in apparently healthy Greenlandic Inuit (Rusiecki et al. 2008). The degree of DNA methylation decreased with increasing concentrations of *p*,*p*′-DDT (dichlorodiphenyltrichloroethane), *p*,*p*′-DDE (dichlorodiphenyldichloroethylene), β-hexachlorocyclohexane, oxychlordane, α-chlordane, mirex, the sum of several poly-chlorinated biphenyl congeners, and the sum of all POPs studied. Even though their concentrations of POPs were much higher than those in other general populations, the global DNA hypomethylation mechanism may be involved in associations between background exposure to POPs and various chronic diseases, including type 2 diabetes, metabolic syndrome, and coronary heart disease (Ha et al. 2007; Lee et al. 2006, 2007).

It is worthwhile to note that some organo-chlorine (OC) pesticides and polychlorinated biphenyls (PCBs) are still used in developing countries such as India or China and that they persist in the food supplies of all countries. In addition, large amounts of municipal wastes are dumped daily in open dumping sites in the suburbs of major cities without proper management. A recent study reported that the surrounding environment showed extremely high levels of POPs compared with those in the control sites (Minh et al. 2006). In fact, *in utero* exposure to various chemical contaminants such as dioxins, PCBs, or OC pesticides may cause adverse pregnancy outcomes such as low birth weight, preterm delivery, or intrauterine growth retardation (Wigle et al. 2008; Windham and Fenster 2008), which are commonly attributed to under-nutrition and a future high risk of metabolic syndrome and type 2 diabetes in developing countries (Yajnik 2004). Epigenetic changes due to POPs may likely play a role in the current epidemics of metabolic syndrome and type 2 diabetes in developing countries, along with westernization of life style. However, our hypothesis may be relevant to any population with background exposure to various chemicals that are conjugated with GSH.

## Conclusions

At present, there is a paucity of integrative human studies that consider both chemicals and nutrition. Therefore, future human studies will be needed to consider the influences of chemical exposures on DNA hypomethylation, accounting for dietary factors.

Especially in times of generally adequate diet, studies of epigenetic change may miss a factor of considerable importance if they do not consider chemical agents. Because epigenetic changes are reversible, developing drugs that control epigenetic regulation now attract substantial research investment, including the development of functional foods or supplements. However, because lifelong exposure to some chemical agents is likely to play an important role in epigenetic changes of etiopathogenic relevance for some adult diseases, it would also be reasonable and efficient to strengthen public and private policies that decrease exposure to such chemicals.
